# LIR Motif-Containing Hyperdisulfide β-Ginkgotide is Cytoprotective, Adaptogenic, and Scaffold-Ready

**DOI:** 10.3390/molecules24132417

**Published:** 2019-06-30

**Authors:** Bamaprasad Dutta, Jiayi Huang, Janet To, James P. Tam

**Affiliations:** School of Biological Sciences, Nanyang Technological University, 60 Nanyang Drive, Singapore 637551, Singapore

**Keywords:** adaptogenic, autophagy, β-ginkgotide, cytoprotective, cysteine-rich peptides, disulfide-rich scaffold, hyperdisulfide, hypoxia, LIR motif, ginkgo nuts

## Abstract

Grafting a bioactive peptide onto a disulfide-rich scaffold is a promising approach to improve its structure and metabolic stability. The ginkgo plant-derived β-ginkgotide β-gB1 is a highly unusual molecule: Small, hyperdisulfide, and found only in selected ancient plants. It also contains a conserved 16-amino-acid core with three interlocking disulfides, as well as a six-amino-acid inter-cysteine loop 2 suitable for grafting peptide epitopes. However, very little is known about this recently-discovered family of molecules. Here, we report the biophysical and functional characterizations of the β-ginkgotide β-gB1 from *G. biloba*. A circular dichroism spectroscopy analysis at 90 °C and proteolytic treatments of β-gB1 supported that it is hyperstable. Data mining revealed that the β-gB1 loop 2 contains the canonical LC3 interacting region (LIR) motif crucial for selective autophagy. Cell-based assays and pull-down experiments showed that β-gB1 is an adaptogen, able to maintain cellular homeostasis through induced autophagosomes formation and to protect cells by targeting intracellular proteins from stress-mediated damage against hypoxia and the hypoxia-reoxygenation of induced cell death. This is the first report of an LIR-containing peptide natural product. Together, our results suggest that the plant-derived β-ginkgotide is cytoprotective, capable of targeting intracellular proteins, and holds promise as a hyperdisulfide scaffold for engineering peptidyl therapeutics with enhanced structural and metabolic stability.

## 1. Introduction

Bioactive peptides, particularly peptide hormones that are small in size and flexible in structure, are useful drug leads. They have the advantages of being amenable to chemical synthesis, multiple derivatizations, combinatorial chemistries, and structure-activity relationship studies. However, they suffer the limitations of receptor selectivity, short serum half-life, and poor oral bioavailability [1,2].

For five decades, Hruby and his co-workers have addressed these limitations using a multi-disciplinary approach, combining structure analysis, pharmacology, and chemistry to achieve the desired bioactivity and receptor selectivity of peptide hormones such as α-, β- and γ-melanotropins, neuropeptides, and opioid peptides. In chemistry, they championed the use of structure-restricting tools through various combinations of backbone or side chain-side chain cyclization. For conformation-restricting tools, they pioneered peptidomimetics and conformationally-hindered amino acids. Their collective works have strongly impacted the design of peptidyl drugs and approaches to medicinal chemistry [1,3,4,5,6].

Nature also provides peptide natural products with various schemes of constraints to enhance their structure and metabolic stability. These schemes, especially features that provide exceptional stability against proteolysis, can be found in two rapidly expanding and highly diverse superfamilies of natural products: The ribosomally synthesized and post-translationally modified peptides (RiPPs) [7] and the cysteine-rich peptides (CRPs) [8,9].

RiPPs are richly represented by the microbial-derived peptides such as microcins, lanthipeptides, and lasso peptides [10]. CRPs are found in both plants and animals. In animals, they are well represented by the ion-channel modulating venom-peptide toxins, which are found in cone snails, insects, and snakes [11,12]. In plants, CRPs are often found as host-defense molecules against environmental stress, pests, and pathogen attacks [8,13].

Over the past 20 years, our laboratory has been interested in the discovery of novel CRPs from medicinal plants—particularly those CRPs with a high potential for oral activity [14,15,16,17,18,19,20,21]. To meet this goal, we have focused our efforts on disulfide-rich or cystine-dense plant peptides containing three or more disulfide bonds with a molecular size of <6 kDa. Such disulfide-rich peptides would have a high structural and metabolic stability because of multiple cross-braces by disulfides. In addition, such peptides would contain, on average, a cysteine in every four or five amino acids in their sequence or about 18–25% cysteine per molecule. Because of their structure and metabolic stability, disulfide-rich peptides have been used successfully as scaffolds to graft bioactive peptides [22,23,24]. 

Recently, we discovered hyperdisulfide peptides from medicinal plants. These peptides contain a cysteine in every three amino acids or about 30% cysteine per molecule. However, plant-derived hyperdisulfide peptides have not been explored as a scaffold for drug design. Their exceptional compact structure suggests that these hyperdisulfide peptides could also be a hyperstable scaffold for engineering peptides to enhance their metabolic stability while retaining their bioactivity. 

*Ginkgo biloba*, commonly known as ginkgo or maidenhair tree, is one of the most ancient tree species. Both the leaves and nuts have been used in traditional medicines [22]. Recently, we have identified two CRP families in *G. biloba* nuts, α- and β-ginkgotides [18,19]. The α-ginkgotides are disulfide-rich and belong to the 8C-hevein-like peptide family. In contrast, the β-ginkgotides are hyperdisulfide peptides which contain a conserved six-cysteine core with a highly clustered cysteine spacing and a motif of C–CC–C–CC, an arrangement that has not been reported in cysteine-rich peptides. Furthermore, we found that the α-ginkgotides display anti-fungal activity, but the bioactivity of β-ginkgotides remains unexplored. 

Here, we report the biophysical and functional characterizations of the hyperdisulfide-constrained the β-ginkgotide β-gB1 from *G. biloba.* We found that β-ginkgotide is highly resistant to heat and proteolytic degradation, suggesting it is a suitable scaffold for grafting peptide epitopes. Bioinformatics data-mining revealed that β-gB1 contains the canonical LIR (LC3 interacting region) motif. Functionally, we showed that the β-ginkgotide β-gB1 displays cytoprotective and adaptogenic properties to modulate cellular homeostasis and survivability against hypoxia-induced stress.

## 2. Results

### 2.1. β-Ginkgotide: Synthesis and Biophysical Characterization

#### 2.1.1. Chemical Synthesis and Oxidative Folding of β-Ginkgotide

**Scheme 1:**
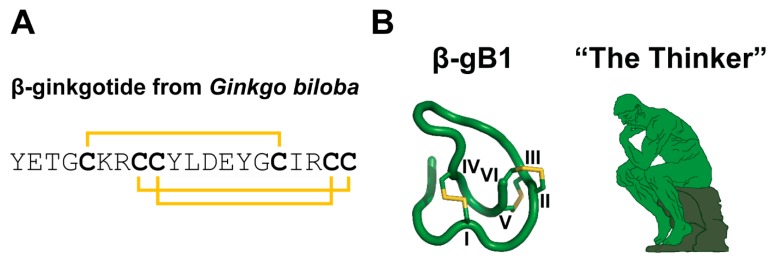
β-ginkgotide, β-gB1 from *Ginkgo biloba*. (**A**) Primary sequence and disulfide connectivities (Cys I-IV, Cys II-VI, and Cys III-V) of β-gB1. (**B**) Cartoon representation of the NMR structure of synthetic β-gB1 (PDB: 5XIV) resembles the Rodin sculpture “The Thinker (Le Penseur).” The central loop 2 (between Cys III and Cys IV, the vicinal cysteine is counted as one cysteine) containing the peptide epitope YLDEYG forms the head and the back of “The Thinker.” Two additional forms of the β-ginkgotide β-gB1 (not shown in the scheme) are: The *S*-reduced form where all six cysteines remained as free sulfhydryls, and the *S*-alkylated form in which the reduced β-gB1 was *S*-alkylated with iodoacetamide.

We prepared three forms of β-ginkgotide β-gB1: The folded form, the *S*-reduced form where all six cysteine residues remained as free sulfhydryls, and the *S*-alkylated form in which the *S*-reduced β-gB1 was *S*-alkylated with iodoacetamide (Scheme 1, legend). A synthetic β-ginkgotide β-gB1 was obtained from GL Biochem (Shanghai, China) by a stepwise solid phase peptide synthesis using Fmoc (*N*-(9-fuorenyl)methoxycarbonyl) chemistry. However, the acidolytic cleavage, HPLC purification, and oxidative folding were performed in our laboratory. The oxidative folding was performed according to the conditions previously reported using a mixture of reduced and oxidized glutathione together with dimethyl sulfoxide in a 75% yield before purification. The mass spectrometry (MS) spectra and HPLC chromatogram of the folded β-gB1 are shown in Supplementary Figure S1. A co-elution test and 2D NMR comparison in our previous study showed that under this folding condition, the folded β-gB1 shared the same peptide fold as the native β-gB1 extracted from *G. biloba* [19].

#### 2.1.2. β-Ginkgotide Secondary Structure and Stability

**Figure 1:**
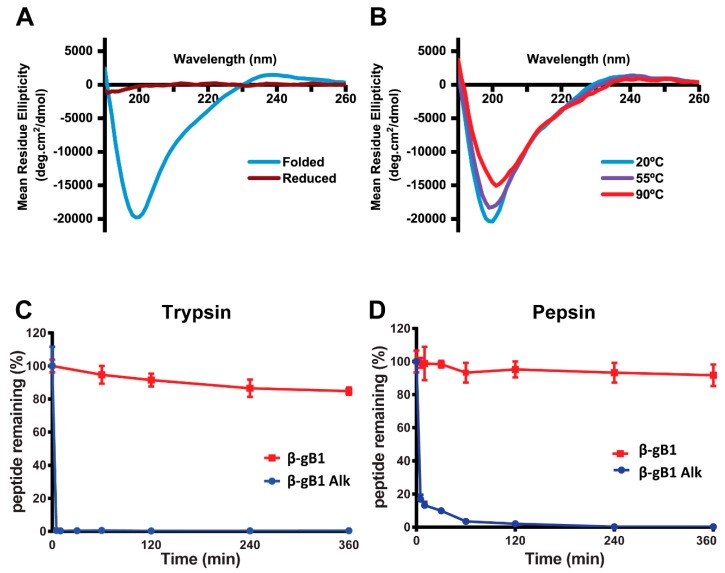
Comparison of the stability of the folded β-gB1 and *S*-reduced and *S*-alkylated forms. (**A**) Far-UV CD spectra of folded and *S*-reduced β-gB1 peptides at 20 °C. (**B**) Far-UV CD spectra of the folded β-gB1 peptide at 20, 55 and 90 °C. Proteolytic stability of β-gB1 against (**C**) trypsin and (**D**) pepsin for six hours in the buffer (pH 7.8) at 37 °C. The *S*-alkylated β-gB1 (β-gB1 Alk) was used as a control.

The secondary structure and thermal stability of the synthetic β-gB1 were assessed using circular dichroism (CD) spectroscopy. The far-UV CD data of β-gB1 were acquired at 20 °C, and secondary structure composition was predicted using the CDSSTR algorithm [23] with a normalized root mean square deviation (RMSD) value of 0.021. The CD spectroscopy spectra agreed with the predicted secondary structure of folded β-gB1, which consists 28% strands, 16% turns, 1% helices, and 55% unordered structures (Figure 1A). The spectrum is in strike contrast with that of the *S*-reduced β-gB1, which is devoid of a secondary structure (Figure 1A). Heating the folded β-gB1 peptide up to 90 °C did not alter its CD spectrum (Figure 1B), thus indicating that this hyperdisulfide peptide is highly resistant to thermal denaturation, which is consistent with our previous observations [19].

To assess the proteolytic stability of β-gB1, it was treated with trypsin or pepsin for 6 h. Figure 1C reveals that about 90% of the β-gB1 remained intact after a six hour trypsin or pepsin treatment. In contrast, the *S*-alkylated β-gB1 was completely degraded after a five minute treatment with trypsin at 37 °C. Similarly, the *S*-alkylated β-gB1 was degraded completely by pepsin within 30 min. Our results suggest that the presence of multiple disulfide bonds is essential for the stability of β-gB1 against proteolytic degradation. This result is also consistent with our previous observations that the hyper-constrained β-gB1 is hyperstable [19]. Prolonging the proteolytic treatment does not change the trend of degradation. This is in agreement with other plant CRPs that contain a compact structure, whose proteolytic stability was examined up to six hours treatment [14,17,18,24].

### 2.2. Intrinsic Functions of β-Ginkgotide 

#### 2.2.1. β-Ginkgotide Contains the LIR Motif and Induces Autophagosomes Formation

We performed a motif search of β-gB1 using the eukaryotic linear motif (ELM) resource and found that the hexapeptide sequence DEYGCI in the loop 2 of β-gB1 (Scheme 1) matches the canonical LC3-interacting region (LIR) motif [25]. The LIR-containing proteins regulate autophagosome formation by interacting with the protein Atg8 (autophagy-related gene, Atg) family through their LIR sequences [26]. Based on this data mining information, we examined β-gB1 in autophagy using the neuronal-like SH-SY5Y cells. The SH-SY5Y cells were cultured with or without β-gB1. The autophagy markers, Beclin-1 (BECN1), LC3B-I, and LC3B-II, were identified and quantified by western blot analysis. Beclin-1 plays an important role in regulating both autophagy and cell death [27], whereas LC3B is an important component of the autophagy pathway and the most widely used marker for autophagosomes. Two of the processed LC3B protein are LC3B-I and LC3B-II. LC3B-I is a truncated LC3B. In contrast, LC3B-II is a truncated and lipidated version of LC3B-I [28,29]. Importantly, LC3B-II is centrally involved in the events of autophagosome membrane expansion and fusion.

**Figure 2:**
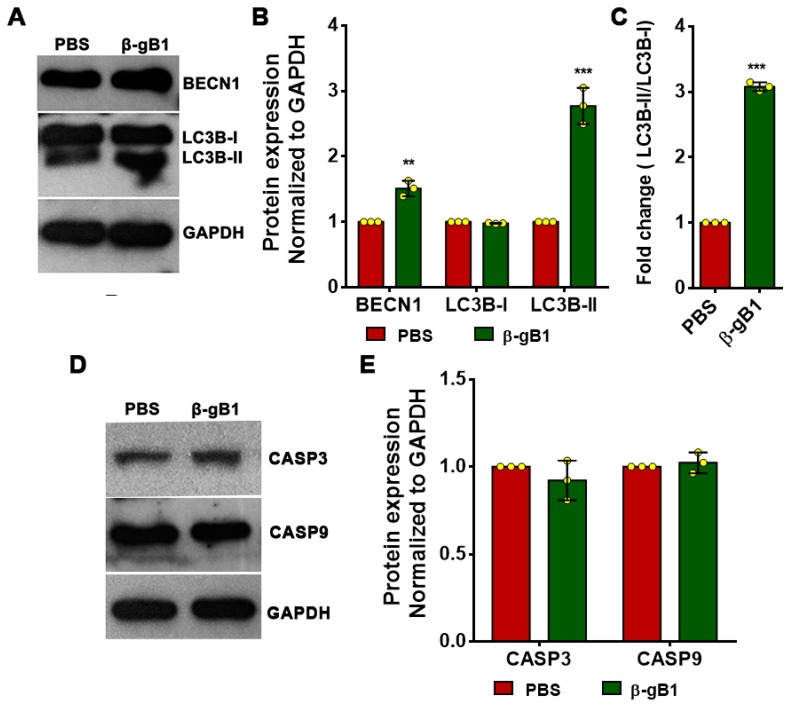
β-ginkgotide-induced autophagy. **A**. Western blot of BECN1, LC3B-I, and LC3B-II in β-gB1-treated and untreated cells. **B**. Expression of BECN1, LC3B-I, and LC3B-II compared with the control. **C**. Ratio of LC3B-II/ LC3B-I upon β-gB1 treatment. **D**. Western blot of caspases CASP3 and CASP9 in β-gB1-treated and untreated cells. **E**. Expression of caspases CASP3 and CASP9 I compared with the control. Quantification was based on western blot band intensity, and data are shown as mean values. Statistical significance was calculated from biological triplicates (** *p* < 0.005 and *** *p* < 0.0005).

Figure 2A,B shows the expression of all three autophagy markers by western blot analysis. The β-gB1 treatment did not alter the expression of the LC3B-I isoform. In contrast, the β-gB1-treated cells increased the expression of the LC3B-II isoform by 2.8-fold. Figure 2C shows that the LC3B-II/LC3B-I ratio increased about 3-fold upon β-gB1 treatment compared to the control. In addition, the β-gB1-treated cells increased the BECN1 expression by 1.5-fold (Figure 2A,B). The elevated expression of both the LC3B-II isoform and BECN1 suggest that β-gB1 induced autophagosomes formation in SH-SY5Y cells. To show that β-gB1 was not involved in apoptosis, the SH-SY5Y cells were treated with β-gB1. Our results showed that the expression of caspase-3 and caspase-9, two enzymes that are critical to the apoptotic pathway, remained unchanged in the treated cells (Figure 2D,E), suggesting that no β-gB1-induced apoptosis occurs. 

#### 2.2.2. β-Ginkgotide Protects Hypoxia-Induced Cell Death

**Figure 3:**
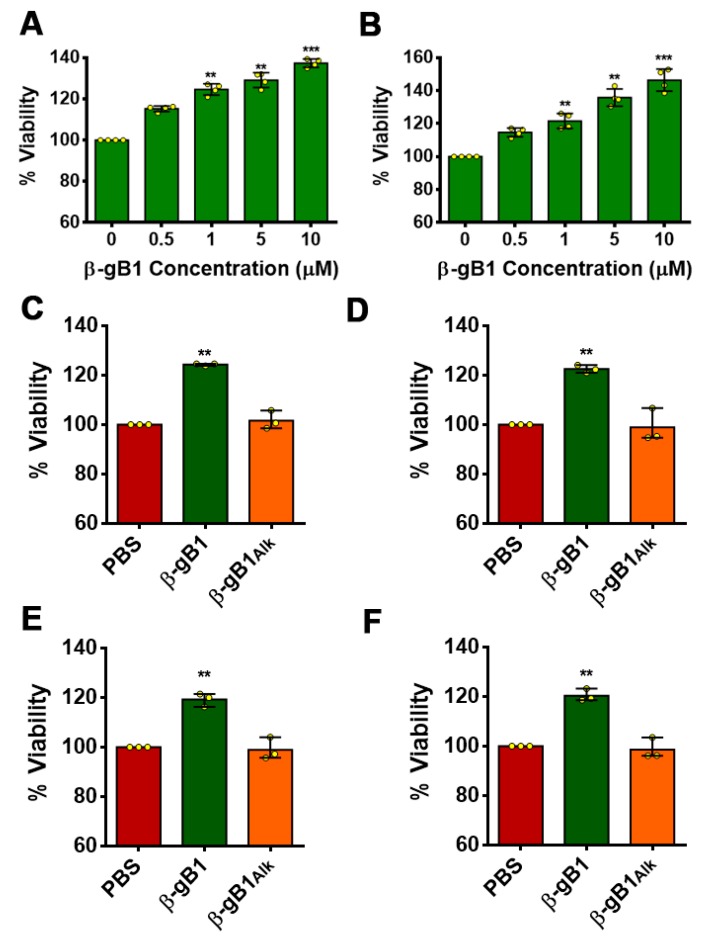
Effect of β-ginkgotide against hypoxia and hypoxia-reoxygenation-induced cell death. Cells were cultured with β-gB1/*S*-alkylated β-gB1 (β-gB1_Alk_, see Scheme 1 legend) under hypoxia/hypoxia-reoxygenation condition for 48h, and viability was measured by an MTT assay. PBS was used as a vehicle control. Dose-response of β-gB1 upon cell survivability of H9c2 (**A**) and SH-SY5Y (**B**) cells under a hypoxic environment. Relative survival of H9c2 (**C**) and SH-SY5Y (**D**) cells upon 1µM β-gB1 treatment during 48h hypoxia. Relative survivals of H9c2 (**E**) and SH-SY5Y (**F**) cells upon 1µM β-gB1 treatment during hypoxia-reoxygenation stress. Viabilities are expressed as mean values, and statistical significance was calculated from three independent experimental replicates. ** *p* <0.005 and *** *p* < 0.0005.

**Figure 4:**
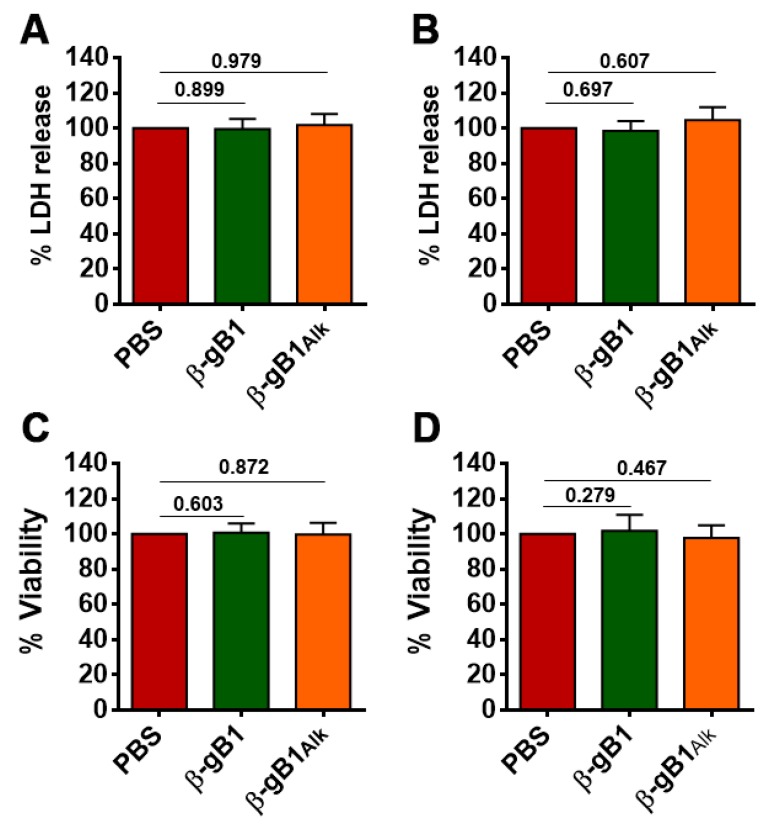
Effect of β-ginkgotide in cell growth and death. The cytotoxicity and plasma membrane-damaging effect was measured by a lactate dehydrogenase (LDH) release assay. Cells were cultured and with or without 10µM of β-gB1/*S*-alkylated β-gB1 (β-gB1_Alk_) for 48 h, and an LDH release was measured by an LDH assay kit. PBS was used as a vehicle control. The Y-axis represents the % LDH release by H9c2 (**A**) and SH-SY5Y (**B**) cells compared to their vehicle control counterpart. Statistical significance was determined from experimental triplicates. Cells were cultured with or without 10µM of β-gB1 and *S*-alkylated β-gB1 (β-gB1_Alk_) for 48 h, and viability was measured by an MTT assay. The relative viability of H9c2 (**C**) and SH-SY5Y (**D**) cells are expressed as mean values, and statistical significance was calculated from independent experimental triplicates.

Autophagy is an integrated stress response that clears the subcellular damages. Previous studies have shown that such damages can be induced by hypoxia and hypoxia-reoxygenation stress. Clearing subcellular damages would increase survivability [30,31,32]. In traditional medicine, *G. biloba* is used as an herbal remedy for acute ischemic stroke and post-stroke recovery [33,34,35]. To examine the effects of β-gB1 in hypoxia stress, we used cardio-myoblast H9c2 and SH-SY5Y cells. Cells were cultured with or without β-gB1 and incubated in a hypoxic environment for 48 h before the assessment of cell survival by the MTT assay. Figure 3A shows that 10 µM of β-gB1 increases H9c2 survivability by 37%. A comparable cytoprotective effect was also observed in SH-SY5Y cells. Importantly, when the concentration of β-gB1 was reduced tenfold from 10 to 1 µM, a 22–24% increase of survivability was still observed in H9c2 and SH-SY5Y cells (Figure 3A–D). In contrast, no cytoprotective effect was observed in cells treated with a control peptide, the linear *S*-alkylated β-gB1 (Figure 3C,D), suggesting that the folded form of β-gB1 is important for the cytoprotective activity. No change in cell viability was observed during a normal oxygenated environment (normoxia) in both β-gB1 and *S*-alkylated β-gB1–treated H9c2 and SH-SY5Y cells (Figure 4C,D), suggesting that β-gB1 or its *S*-alkylated form has no effect on cell proliferation and cell death. Collectively, these results confirm the cytoprotective effects are exerted by the folded hyperdisulfide form but not the *S*-alkylated form of β-gB1. 

#### 2.2.3. β-Ginkgotide Protects Cells from Hypoxia-Reoxygenation Injury 

The cytoprotective effect of β-ginkgotides against hypoxic stress led us to examine the effect of β-gB1 under hypoxia-reoxygenation stress. Cells were cultured with or without β-gB1 and its *S*-alkylated β-gB1, first under the hypoxic condition for 24 h and then at a normal oxygen environment for another 24 h. The cell viability under hypoxia-reoxygenation condition was assessed by the MTT assay. Upon β-gB1 treatment, cell survivability was increased by 24% and 19% in H9c2 and SH-SY5Y cells, respectively (Figure 3E,F). In contrast, the *S*-alkylated β-gB1 did not produce any cytoprotective effect against hypoxia-reoxygenation injury (Figure 3E,F). Again, our results confirm the importance of the folded structure of β-gB1 against hypoxia-reoxygenation injury.

#### 2.2.4. β-Ginkgotide in Cytotoxicity

Previously, we showed that β-gB1 does not exhibit an antimicrobial effect against *Escherichia coli*, *Candida albicans,* and *Candida tropicalis* at concentrations up to 100 µM. In addition, β-gB1 is non-toxic to Vero and BHK (baby hamster kidney) cell lines based on an MTT assay [19]. To show that β-gB1 is membrane-permeable to target intracellular proteins that induce autophagy formation and cytoprotective against hypoxia, we used a lactate dehydrogenase (LDH) release assay to test the non-toxic effect of β-gB1 in H9c2 and SH-SY5Y cells. LDH is a cytoplasmic enzyme which is rapidly released into the cell culture medium upon plasma membrane damage and is widely used as a marker for cytotoxicity. Thus, an LDH-release assay measures the level of plasma membrane damage to a cell population. Indeed, an LDH-release assay showed that 10 µM of β-gB1 or the *S*-alkylated β-gB1 treatment produced no significant cytotoxicity or cell-membrane damaging effects on both cell lines (Figure 4A,B). An MTT-based cell proliferation assay showed that treatment of 10 µM of β-gB1 or *S*-alkylated β-gB1 has no significant effect on the growth or death of H9c2 and SH-SY5Y cells (Figure 4C,D). Together, our experimental data suggest that both the linear *S*-alkylated and folded forms of β-gB1 have no cytotoxic effect on our tested cell lines in the 10 µM dose range.

## 3. Discussion

This report describes, for the first time, that the hyperdisulfide peptide, the β-ginkgotide β-gB1 derived from the ginkgo plant, is cytoprotective against hypoxia stress. It also acts as an adaptogen because it induces autophagy, an adaptive process that maintains cellular homeostasis and promotes cell survival. Overall, our findings suggest that the observed cytoprotective and adaptogenic effects of β-ginkgotide agree with the ethnomedicinal uses of ginkgo for treating ischemic stroke, post-stroke recovery cognitive disorders, and Alzheimer’s disease [22,23,24]. The following discussion further amplifies the significance and salient features of this highly unusual molecule. 

Autophagy, a degradation process for macromolecules and organelles, maintains homeostasis in cells and tissues. Historically, autophagy has been viewed solely as a degradation process to replenish the nutrition and energy of cells during starvation. However, recent research has described various forms of selective autophagy which have relevance to the treatment of various pathological conditions which include cancer, neurodegenerative diseases, muscular dystrophy, ageing, and innate immunity [36].

A major driving force for this change of view is the discovery of specific autophagy receptors that sequester cargo into forming autophagosomes (phagophores) [26]. In turn, these double-membraned phagophores merge with lysosomes for degradation. The key to this selectivity is the LC3-interacting region (LIR) motif, which guards and locks the targeting of autophagy receptors to LC3 or other ATG8 family proteins anchored in the phagophore membrane. LIR-containing proteins include a complex array of receptors, proteins associated with basal autophagy apparatus, vesicles formation and their transporters, GTPase activating proteins, and specific signaling proteins [26].

**Scheme 2:**
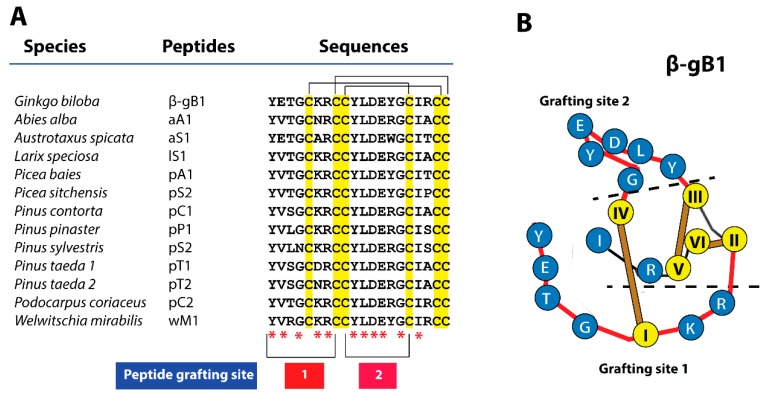
Sequences and the suitable grafting sites of β-ginkgotide and β-ginkgotide-like peptides. (**A**) Sequence comparison between the β-ginkgotide β-gb1 and twelve selected β-ginkgotide-like peptides with highly conserved sequences. The two suitable grafting sites, 1 and 2, are indicated. (**B**) The schematic representation of the grafting sites 1 and 2 are colored in red. * Represents highly and absolutely conserved residues.

The LIR-containing proteins which interact with ATG8 contain a consensus sequence **W/F/Y**-X-X-**L/I/V**, a sequence motif with two essential hydrophobic amino acids (in bold) separated by two amino acids [37]. This LIR motif is often preceded by two acidic amino acid residues to form a six-amino-acid recognition sequence that fits tightly into the basic and hydrophobic pocket on ATG8, the LIR-docking site (LDS). Thus, the six-amino-acid LIR is the minimal required sequence to interact with the Atg proteins. The loop 2 sequences of β-gB1 and β-gB1-like peptides from different ancient plants are highly conserved. In particular, some contain the consensus LIR motif DEYGCI (Scheme 2), suggesting that they could play a role in autophagy [38]. 

The conversion of the LC3-I isoform (soluble form of LC3) to the lipidated LC3-II isoform is an essential step of autophagosome formation [39,40]. The LC3-II isoform is stably associated with the autophagosome and is widely used as an essential indicator of autophagosome formation [40]. We show that the β-ginkgotide β-gB1 increases the expression of the lipidated LC3B-II isoform. The autophagosome will fuse with an available lysosome as a special waste management and disposal process for degradation of the toxic protein aggregates, implicated in the neurodegenerative diseases [36,39]. In addition, we showed that BECN1 expression, a key pathogenic factor for the pathogenesis of neurodegenerative diseases including Alzheimer’s disease, is elevated, and the overexpression of BECN1 has been found to reduce disease pathogenesis through the autophagy-mediated clearance of the toxic amyloid precursor [33].

As a part of the integrated stress response to maintain cellular homeostasis which increases cellular survivability, autophagy involves the clearing of the subcellular damages induced by environmental stress, including hypoxia and hypoxia-reoxygenation stress [40,41,42]. Thus, the autophagy-inducing property of β-gB1 could be beneficial for minimizing the pathogenic effects of different environmental stresses including hypoxia. Resected circulation reduces the oxygenation levels in tissues, a condition which is commonly known as hypoxia [19]. The restoration of circulation after hypoxia results in hypoxia-reoxygenation stress, resulting in the alternation of several important pathways including ion channels, reactive oxygen species, inflammation, and endothelial dysfunction, followed by apoptosis-induced cell death and tissue damage [30,31,43]. Those two adverse physical factors are the key mediators for the pathogenesis of diseases including ischemic heart diseases, stroke, and cancer [30,31,32,43,44,45,46,47]. Thus far, the limited knowledge and availability of the therapeutics has complicated the treatment against hypoxia-induced pathogenesis. In this regard, our results are promising in showing the adaptogenic and cytoprotective effects of the β-ginkgotide β-gB1, effects which could minimize hypoxia- and hypoxia-reoxygenation-induced pathogenesis and increase cell survivability in cardiomyocyte and neuronal cells. 

The interactions of β-gB1 with the intracellular protein targets in autophagy and hypoxia pathways suggest that it is membrane permeable. These data, coupled with our results, show that the cell membranes are not damaged based on an LDH release assay. In addition, we previously showed that β-gB1 is not a membranolytic antimicrobial [19]. Furthermore, our data have shown that the linear *S*-alkylated form of β-gB1 is not cytoprotective or adaptogenic, strongly suggesting that the folded and structurally compact β-gB1 is necessary for membrane permeability to interact with its intracellular targets. 

A distinguishing feature of the β-ginkgotide β-gB1 is its high amount of cysteine residues. The intercysteinyl distance of the hyperdisulfide β-gB1 is relatively small, with 16 residues between the first and six cysteine residues, accounting for 38% of cysteine. In contrast, the average cysteine content of most plant six-cysteine-containing CRPs contain 17–27% of cysteine [19].

Scheme 2 compares the sequences of β-ginkgotide with 12 β-ginkgotide-like peptides, and it shows that they are highly conserved with 80% sequence identity. Two suitable epitope grafting sites can be envisioned. Site 1 is the *N*-terminus, whereas site 2 is the longer and highly conserved loop 2 with six amino acids. If necessary, the four residues of the amino terminus of site 1 can be combine with site 2 for grafting even longer peptides. 

Table 1 compares the cysteine frameworks of the β-gB1 with three hyperdisulfide peptides from the conotoxin family, the scratch peptide, BtIIIA, and Mr3e (ConoServer, the database of conotoxins (http://www.conoserver.org)). From the standpoint of grafting a peptide epitope, β-gB1 would be the most suitable. First, it contains the longest interdisulfide loop among the selected hyperdisulfide peptides, loop 2 with six amino acids, which is highly amenable for grafting peptides. In contrast, the other three hyperdisulfide peptides listed in Table 1 contain less than four amino acids in their intercysteinyl loops. Secondly, the intrinsic property of β-gB1 is cytoprotective, which makes it more desirable than a toxin derived from the conotoxin family. Thirdly, β-gB1 is membrane permeable and could target intracellular proteins. Overall, our results suggest that the β-ginkgotide β-gB1 is a novel hyperdisulfide scaffold that is suitable for grafting bioactive peptides to improve their structures and metabolic stability.

## 4. Materials and Methods

### 4.1. Reagents

All reagents were purchased from Sigma-Aldrich unless otherwise indicated (Sigma-Aldrich, Madison, WI, USA). Antibodies against GAPDH (6C5) (sc-32233) were from Santa Cruz Biotechnology, Santa Cruz, CA, and the Anti-LC3B (L7543) antibody was purchased from Sigma-Aldrich, WI. Beclin-1 (D40C5) (3495), Caspase-3 (9662), and Caspase-9 (9502) antibodies were procured from Cell Signaling Technology, Danvers, MA. Clarity Max™ Western ECL blotting substrates (1705062) were purchased from Bio-Rad Laboratories, Segrate, Italy.

### 4.2. Synthesis and Oxidative Folding of β-Ginkgotide β-gB1

A synthetic β-ginkgotide β-gB1 was obtained from GL Biochem (Shanghai, China) by solid phase peptide synthesis using Fmoc (*N*-(9-fluorenyl) methoxycarbonyl) chemistry and purified by C_18_-reversed-phase HPLC to 85% purity. The oxidative folding was performed and modified as previous report [19]. Briefly, the oxidative folding was initiated by adding 1 mg of synthetic, reduced β-gB1 to 1 mL of a 0.1 M ammonium bicarbonate buffer (pH 8.5) containing 20 mM of reduced glutathione, 4 mM of oxidized glutathione and 10% (*v*/*v*) dimethyl sulfoxide (DMSO). After a 2-h incubation, the reaction was quenched 0.1% trifluoroacetic acid solution and purified by C_18_-reversed-phase HPLC.

### 4.3. Circular Dichroism Spectroscopy

Far-UV CD spectra were recorded for the synthetic and folded β-ginkgotide β-gB1 (42 µM) in a sodium phosphate buffer (pH 7.5) on a Chirascan™ CD spectrometer (Applied Photophysics, Leatherhead, UK) equipped with a temperature controller. Spectra were acquired between 190 and 260 nm from 20 to 90 °C using a 1 mm path-length quartz cuvette, a 1 nm spectral bandwidth, and a 1 nm step size. CD spectroscopy data were analyzed for secondary structure content using CDSSTR [23]. CD data were expressed as mean residue ellipticity (in deg cm^2^dmol^−1^).

### 4.4. Enzymatic Stability

The folded β-gB1 (28 µg) was added to 100 μL of a 0.1 M ammonium bicarbonate buffer (pH 7.8) containing 800 U/mL of trypsin or 4 mg/mL of pepsin and incubated at 37 °C for 6 h. At each time-point (0, 1, 2, 4, and 6 h), 20 µL of the treated sample was aliquoted and quenched by adding 5 µL of 1 M hydrochloric acid or 0.8 μL of 1 M sodium hydroxide. The reduced β-gB1 was alkylated with iodoacetamide (*S*-alkylated β-gB1) and used as a control in this study. The reaction mixture was analyzed by MALDI-TOF MS and C_18_-reversed-phase HPLC to determine its stability.

### 4.5. Cell Culture

Rat cardio-myoblast cells (H9c2) and mouse neuron-like SH-SY5Y cells were purchased from ATCC and maintained in DMEM (Dulbecco’s modified eagle medium) medium supplemented with 10% fetal bovine serum (FBS), 10,000 U/mL of penicillin, and 10,000 µg/mL of streptomycin at 37 °C, 5% CO_2_.

### 4.6. Hypoxia and Hypoxia-Reoxygenation Model

Cells were seeded in a cell culture medium with/without β-gB1 and incubated at 37 °C, 5% CO_2_ overnight. The next day, the cells were locked inside a BD GasPak EZ anaerobe pouch system (BD Diagnostics, MD) and incubated at 37 °C for the next 48 h for the hypoxia condition. In addition, the cells were cultured under a normal oxygenated environment for the normoxic control. In hypoxia-reoxygenation experiments, the cells were incubated for 24 h in hypoxic conditions before being incubated for another 24 h under normoxia.

### 4.7. MTT Assay

Cells were cultured in DMEM with or without β-gB1/*S*-alkylated β-gB1 (β-gB1_Alk_) for 48 h under normoxia, hypoxia, and hypoxia-reoxygenation conditions. Phosphate-buffered saline (PBS) was used as a vehicle control. After 48 h, MTT (3-(4,5-dimethylthiazol-2-yl)-2,5-diphenyltetrazolium bromide) reagent was added with a final concentration of 0.5 mg/mL, and it was incubated at 37 °C for 2 h. The culture medium was then aspirated, and purple formazan crystals were dissolved in DMSO for colorimetric quantification at 570 nm using a microplate reader with a reference wavelength of 630 nm. Experiments were performed in triplicates, and statistical calculations and bar graphs were done using GraphPad Prism software (GraphPad Prism version 6.01, La Jolla, California, USA). Data are presented as means ± SD, and multiple comparisons were performed by one-way ANOVA. *P*-values of less than 0.05 were considered significant.

### 4.8. LDH Assay

An LDH release assay was performed using the CytoSelect™ LDH cytotoxicity assay kit (CBA-241, Cell Biolabs, Inc., San Diego, CA, USA). In brief, cells were cultured in DMEM with or without 10 µM of β-gB1 and *S*-alkylated β-gB1 (β-gB1_Alk_) for 48 h at 37 °C and 5% CO_2_. PBS was used for the vehicle control experiment. The culture medium was transferred and mixed with the LDH assay reagent in a ratio of 9:1. The reaction mixture was incubated at 37 °C for 2 h, and colorimetric quantification was performed using a 450 nm wavelength. The assay was performed in experimental triplicates, and statistical calculations and bar graphs were done using GraphPad Prism software (Version 6.01). Data are presented as means ± SD, and multiple comparisons were performed by one-way ANOVA. *P*-values pf less than 0.05 were considered significant.

### 4.9. Western Blot Analysis

A 25 µg equivalent quantity of each protein samples were resolved on a 10% and 15% SDS-PAGE gel accordingly and transferred onto a nitrocellulose membrane. Immunoblotting was performed using anti-protein antibodies against glyceraldehyde 3-phosphate dehydrogenase (GAPDH) (1:5000), LC3B (1:1500), Beclin-1 (1:1500), Caspase-3 (1:1500), and Caspase-9 (1:1500). Blots were developed with Clarity Max Western ECL blotting substrates. Protein bands were quantitated densitometrically using ImageJ software (ImageJ15.0b, Wayen Rasband, NIH, Bethesda, Maryland, USA), expressed relative to GAPDH, and normalized to the vehicle control cells. Statistical calculations and bar graphs were done using GraphPad Prism software (Version 6.01), and three individual experimental data sets were used for the calculation. Data are presented as means ± SD and compared by the Holm–Sidak multiple *t*-tests method. P-values pf less than 0.05 were considered significant.

### 4.10. Bioinformatics Analysis

A short linear motifs (SLiMs) search was performed using the online bioinformatics tool the eukaryotic linear motif (ELM) resource (http://elm.eu.org) [25]. The sequences of β-ginkgotide-like peptides were obtained from the GenBank database as previously described [19].

## Figures and Tables

**Table 1:** Comparison of β-gB1 with three hyperdisulfide conotoxin peptides.

Peptides	Cysteine motif ^1^	No. of aa ^2^	Cys (%) ^3^
Scratch peptide	C^I^C^II^••C^III^••C^IV^••C^V^C^VI^	12	50
BtIIIA	C^I^C^II^•••C^III^••C^IV^••C^V^C^VI^	13	46
Mr3e	XC^I^C^II^••••C^III^•••C^IV^•C^V^C^VI^X	14	43
β-gB1	X_4_CI••C^II^C^III^••••••C^IV^••C^V^C^VI^	16	38

^1^ The red dots represent the amino acids in the longest intercysteinyl loop suitable for grafting peptide epitopes. ^2^ Number of amino acids (aa) within Cys I and Cys VI; ^3^ Cys (%) = (number of Cys/total number of amino acids between Cys I and Cys VI) × 100.

## Supplementary Materials

The following are available online, Figure S1: Mass spectrometry and HPLC Spectrum of folded β-gB1.
