# Supplementary material for: LIR Motif-Containing Hyperdisulfide β-Ginkgotide is Cytoprotective, Adaptogenic, and Scaffold-Ready
Source: Molecules. 2019 Jun 30;24(13):2417. doi: 10.3390/molecules24132417 (PMC6651024; doi:10.3390/molecules24132417)
Supplement: Supplementary file 1 [file molecules-24-02417-s001.pdf]

molecules

MDPI

Supplementary materials for

LIR Motif-Containing Hyperdisulfide β-Ginkgotide is Cytoprotective, Adaptogenic and Scaffold-Ready

Bamaprasad Dutta†, Jiayi Huang†, Janet To and James P Tam*

School of Biological Sciences, Nanyang Technological University, 60 Nanyang Drive, Singapore 637551
*Correspondence: jptam@ntu.edu.sg; Tel: +65-6316 2833
† These authors contributed equally to this work.

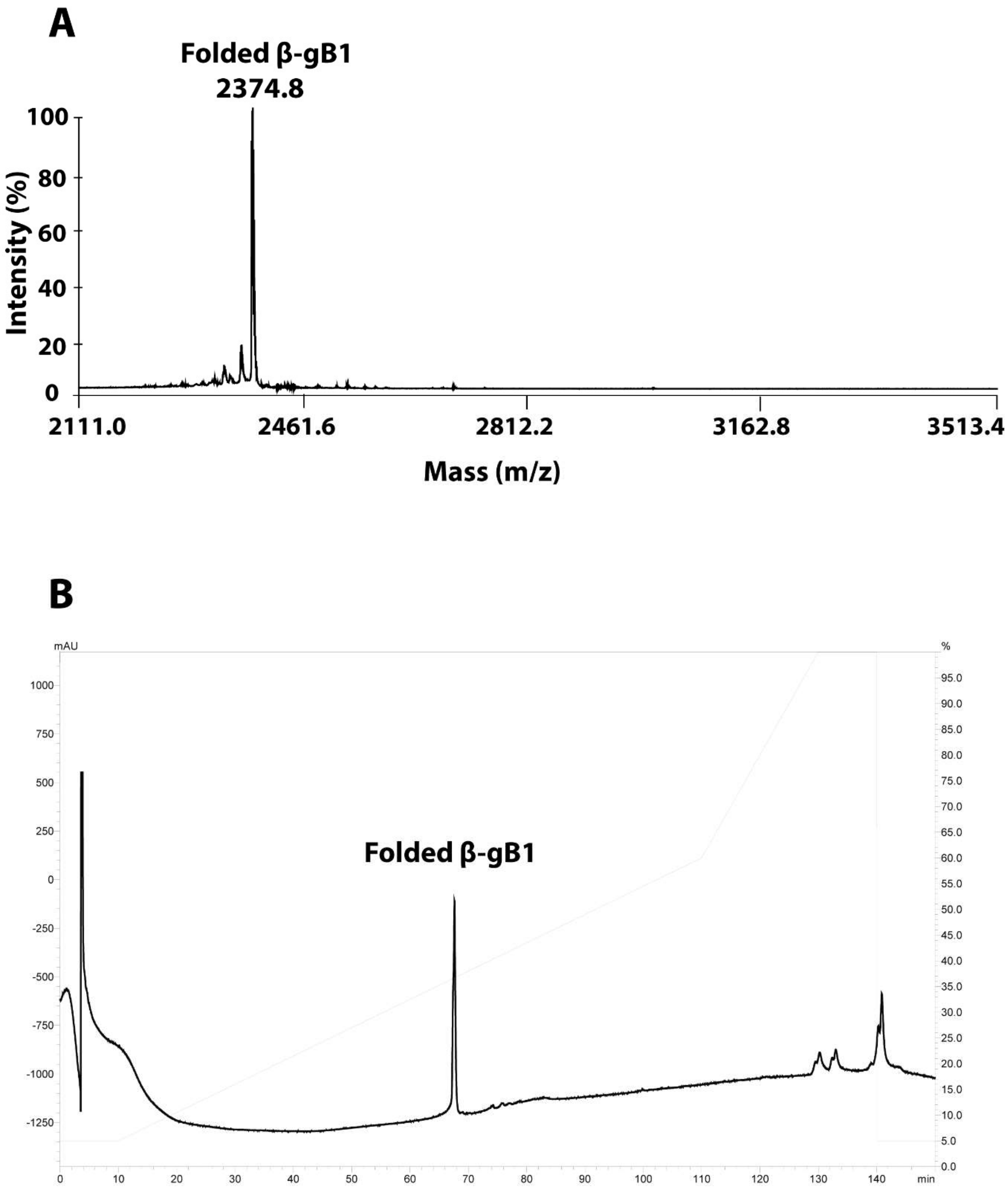

Figure S1. Mass spectrometry and HPLC Spectrum of folded β-gB1. (A) MS spectra of folded β-gB1. (B) HPLC chromatogram of folded β-gB1.

© 2019 by the authors. Submitted for possible open access publication under the terms and conditions of the Creative Commons Attribution (CC BY) license (http://creativecommons.org/licenses/by/4.0/).
